# The Effect of Pulmonary Rehabilitation on Respiratory Functions, and the Quality of Life, following Coronary Artery Bypass Grafting: A Randomised Controlled Study

**DOI:** 10.1155/2021/6811373

**Published:** 2021-09-17

**Authors:** Zümrüt Girgin, Yeliz Ciğerci, Fatıma Yaman

**Affiliations:** ^1^Health Care Service, Afyonkarahisar State Hospital, 03200 Afyonkarahisar, Turkey; ^2^Department of Nursing, Faculty of Health Science, Afyonkarahisar Health Science University, 03200 Afyonkarahisar, Turkey; ^3^Department of Physical Medicine and Rehabilitation, Faculty of Medicine, Kütahya Health Science University, 43100 Kütahya, Turkey

## Abstract

**Objective:**

Examining the effects of a pulmonary rehabilitation (PR) program applied to patients undergoing coronary artery bypass grafting (CABG) surgery with open heart technique on respiratory functions, functional capacity, and quality of life (QoL).

**Design:**

This randomised controlled study applied the Consolidated Standards of Reporting Trials statement.

**Methods:**

The study was conducted with two groups: the intervention group (*n* = 25) and the control group (*n* = 25). The control group received standard care after coronary artery bypass grafting. On the contrary, the experimental group participated in a PR program created by the researchers in addition to standard care. After coronary artery bypass grafting, the respiratory functions (on the 4th day of clinical care) and QoL (at the 6^th^ week) of both groups were evaluated. The primary outcome measure was the respiratory function (forced expiratory volume in one second (FEV1), forced vital capacity (FVC), and FEV1/FVC). The secondary outcome measure of this study was the QoL.

**Results:**

When the average pulmonary function test values of the patients were examined, the mean FVC and FEV1 values of the patients in the intervention group on the 4th day of clinical care were significantly higher with a medium level size effect than the control group (*p* = 0.027; effect size (*d*) = 0.643; *p* < 0.024; effect size (*d*) = 0.658, respectively). At the postoperative 6th week measurement of QoL, a decrease was observed in all subdimensions of the scale, albeit less in the intervention group (*p* < 0.05). There was a decrease in both the mental and physical component summary of QoL (*p* < 0.05).

**Conclusion:**

The results of this study revealed that pulmonary rehabilitation applied to patients who have undergone coronary artery bypass graft recover their functional capacity faster.

## 1. Introduction

Coronary artery bypass grafting (CABG) surgery is aimed at reducing the symptoms related to coronary artery disease, preventing possible complications, and improving the quality of life (QoL) of the patients. On the other hand, CABG is a major surgery that might lead to vital complications [1–5]. Postoperative pulmonary complications (PPCs), a well-reported group of complications following cardiac surgery, are associated with a 4-time increase in mortality, extended intensive care unit and hospital stay, and over $20,000 institutional cost per case [6]. In the cardiac surgery population, measurable derangements in pulmonary function occur in almost all patients, and approximately 10–25% develop PPCs requiring substantial healthcare resource utilization [6]. Cardiopulmonary bypass (CPB), mechanical ventilation, and surgical manipulation of the thoracic cavity each have important roles in the evolution of pulmonary injury [6]. PPCs after cardiac surgery include cardiogenic pulmonary edema, acute respiratory distress syndrome, pneumothorax, pleural effusion, atelectasis, pneumonia, prolonged mechanical ventilation, and phrenic nerve injury [7].

Preventing pulmonary complications that may develop after CABG surgery is important [1, 8, 9]. Pulmonary rehabilitation (PR) is known to be beneficial in preventing and/or reducing pulmonary complications, increasing patient's participation in his/her care, assisting the patient to return to his/her functional life actively, reducing the use of health services afterward, and improving the QoL [10]. Thus, PR is recommended to be included in the standard care of patients undergoing CABG [10–12]. PR is based on preoperative patient training, smoking cessation, deep breathing/cough exercises, lower-upper extremity exercises, the use of incentive spirometer, and early postoperative mobilization [9, 13]. The techniques used in PR may vary depending on the country and the practices of each clinic [14]. Studies conducted on patients who have had CABG surgery have shown that PR significantly improved respiratory muscle strength and function of lungs [13, 15], had a positive effect on the quality of respiratory performance [12], and was effective in reducing surgical morbidity and the cost of medical care [16].

The PR program developed specifically for the patients during the surgical process is a multidisciplinary treatment method that includes a lot of other methods in itself [17, 18]. When compared to other health care professionals, nurses, members of the multidisciplinary team, have the opportunity to observe the patients continuously. Therefore, they have better chances to notice the changes in patients at the early stages [19–21]. They monitor vital signs, changes in airway obstruction, and identify the complications that develop and provide the necessary equipment and patient care [22]. Within the scope of legal regulations in the field of nursing in Turkey, planning, implementing, and evaluating appropriate nursing activities to solve patients' respiratory problems and basic interventional practices such as aspiration, oxygen therapy, body positions, and postural drainage defined the duties, authorities, and responsibilities of nurses, and nurses are expected to implement these applications [23]. Because of these characteristics, they hold a very unique position on the management of the symptoms and providing optimal care for the patient [21]. Furthermore, it is a known fact that there is a direct correlation between the quality of care provided by nurses and patient outcomes [4, 20, 24].

CABG surgery is a procedure that is aimed at improving the QoL of the patient [3, 18, 25]. However, after the surgery, patients undergo a period of recovery that is associated with negative psychological and physical functions that continue for some time [1, 8, 18, 26]. Therefore, integration of the PR program into patient care after the CABG surgery is thought to help achieve the goal of improving the QoL of patients which is the common goal for both PR and the CABG procedure. However, in the literature, there were not enough studies that examined the effects on the respiratory functions and the QoL focusing on the methods together like deep breathing/coughing exercises, mobilization-extremity exercises, inspiratory muscle training, and the use of incentive spirometer. Therefore, the need for this study arose.

### 1.1. Objective

This study is aimed at examining the short-term effects of PR on the respiratory function, functional capacity, and quality of life of the patients undergoing CABG surgery conducted with the on-pump technique.

## 2. Materials and Methods

### 2.1. Study Design

**Figure 1:**
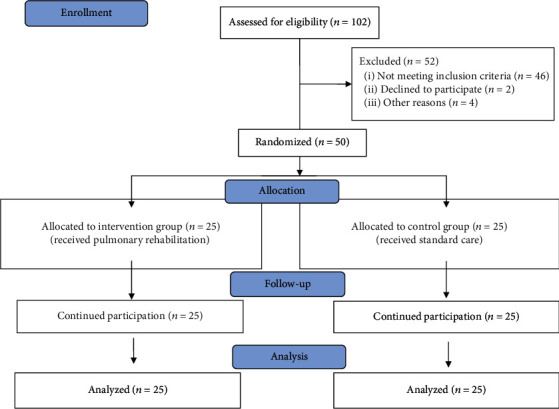
Randomised controlled study flowchart.

This single-centre, randomised controlled trial was performed in the Cardiovascular Surgery Department of Park Hayat Hospital in the Afyonkarahisar province of Turkey. It was conducted between August 2016 and December 2017. This article follows the guidelines for reporting randomised trials (Figure 1).

### 2.2. Participant Selection

#### 2.2.1. Inclusion Criteria

The study involved patients with the following criteria: (a) 18 years old and above; (b) had undergone CABG surgery through the open-heart method; (c) intubated within twenty-four hours; and (d) willing to participate in the study.

#### 2.2.2. Exclusion Criteria

Reasons for exclusion included (a) having orthopedic, psychiatric, and neurological problems; (b) having any comorbid lung disease; (c) had been reoperated on; (d) having postoperative cardiac dysfunction; and (e) had developed postoperative atrial fibrillation.

### 2.3. Setting

#### 2.3.1. Sampling

The sample size in the study was determined using the G. Power 3.1.9.2 program. Sample size estimation was performed based on the expectation of moderate changes (Cohen *d* = 1.14) in pulmonary function test scores (forced expiratory volume (FEV1)/forced vital capacity (FVC)) [27] at postoperative between any groups and control (*α* = 0.05, 1 − *β* = 0.80), and the minimum sample size was estimated at 14 for each group.

#### 2.3.2. Randomization

The patients were divided into two groups (the intervention group and the control group), with 25 patients in each group. The allocation of the patients into the groups was done through the randomization method. Patients were asked to select a piece of paper from a bag with the names of the groups written on it randomly. The patients were allocated into the selected groups, and then they were provided with a verbal briefing about the procedures to be performed in their group. Blinding could not be done in this study.

### 2.4. Measures

The data were collected by using a Sociodemographic Questionnaire Form and Patient Follow-up Form. The sociodemographic questionnaire form included 17 questions to determine the demographic characteristics of the patients. The form was completed during a face-to-face interview on the first day of the patient's admission to the clinic during the preoperative period by asking the questions to the patients. The patient follow-up form was developed to determine the preoperative and postoperative hemodynamic parameters, hemogram, biochemistry, coagulation, blood gas, and Pulmonary Function Test (PFT) parameters, 6 Minute Walk Test (6MWT) parameters, EuroSCORE, Acute Physiological, and Chronic Health Assessment II (APACHE II), and QoL (SF-36).

The EuroSCORE mortality risk assessment system was used to determine mortality risk. EuroSCORE was developed between 1995 and 1999 to determine the projected risk of operative mortality for patients (Roc = 0.76) [28, 29]. Furthermore, the Turkish Social Security Agency mandates the use of the “EuroSCORE mortality risk assessment system” for mortality risk assessment of heart surgery applications for Turkish adults. The EuroSCORE mortality risk assessment was applied to patients during the preoperative period.

The APACHE II scoring system was used to determine the severity, progression, and mortality rate of the disease. The APACHE II scoring system was developed by Knaus et al. (1985) [30]. Acute physiological assessment which is the most important part of the system, age assessment, and chronic health assessment is involved in the system [31]. APACHE II scoring was performed during the patients' first 24-hour period in intensive care.

#### 2.4.1. Primary Outcomes

The Pulmonary Function Test (PFT) is a diagnostic criterion that is used to identify patients with or at risk of pulmonary dysfunction [32]. The PFT assessment was performed by using the NDD Easy on-PC spirometer. During this test, forced expiratory volume (FEV1), forced vital capacity (FVC), and FEV1/FVC ratio at the first second were measured. The PFT was performed by a physician during the preoperative period and on the 4^th^ day of clinical care in the Chest Diseases Outpatient Clinic of the hospital in which the study was conducted.

The 6MWT measures the submaximal level of functional capacity. It was developed by Balke in the early 1960s [33, 34]. The 6MWT was applied to the patients during the preoperative period as well as on the 1^st^ and 4^th^ days of the clinical care. A 30-meter long corridor with a hard and flat surface was chosen for the test. The start and endpoints of the test were marked with a colored token. The patient was advised not to talk to anyone during the test, walk at a normal pace, stop, and rest when feeling tired. Systolic and diastolic blood pressure, SPO2 value, and the respiratory rate of patients were recorded 10 minutes before the test started and at the end of the test. Perception of shortness of breath, as well as fatigue, was determined using a 10-point modified Borg scale. The distance that patients walked at six minutes and how many seconds they rested were recorded on the follow-up form.

#### 2.4.2. Secondary Outcome

The Short-Form Health Survey (SF-36) was used to determine the QoL. The Scale was developed by Ware et al. (1993) (correlation coefficient = 0.43 − 0.90) [35]. The scale, consisting of 36 questions, has 9 subdimensions that measure physical function and role difficulty, emotional role difficulty, social function, mental health, pain, energy, general health perception, and change compared to the previous year. Each subscale's score is recorded individually. The score value was from 0 to 100; a score of 0 indicated the poorest health condition and a score of 100 indicated the best health condition. The SF-36 was applied to the patients during the preoperative period and 6 weeks after the operation when they came to the hospital for a check-up. SF-36 also has two summary scales, a physical component summary (PCS) and a mental component summary (MCS) [36]. PCS and MCS scores, continuous variables in the range of 0 to 100, reflect a patient's overall physical and mental health status, respectively. Higher scores obtained from them indicate better health.

### 2.5. Intervention

In this study, carried out with two groups, the patients who received standard health care services formed the control group, and the patients who underwent PR formed the intervention group. Operations of all the patients admitted to the Cardiovascular Surgery Clinic and included in the study were carried out by the same surgical team. All patients were treated in single rooms, in the Cardiovascular Surgery Clinic of the hospital, where the study was conducted. In the preoperative period, the Cardiovascular Surgery Clinic team briefed all the patients about CABG treatment and its complications, and how long they would stay in intensive care and that their bodies would have drains and catheters. Patients meeting the inclusion criteria were interviewed after their admission to the clinic, one day before surgery, in their rooms. The researcher provided a briefing on the study protocol to patients. The participation of the patients was ensured voluntarily after the researcher briefed the patients about the aim and the protocol of the study. Data for the PFT, 6MWT, EuroSCORE mortality risk assessment, and SF-36 included in the Sociodemographic Questionnaire Form and Patient Follow-up Form were obtained during the preoperative period of the patients in both of the groups. All patients were visited within the first 24 hours of intensive care after CABG. Their APACHE II scores were assessed and recorded. At the same time, the PR program was applied to intervention group patients. PR was applied to intervention group patients for four days starting from the day they were transferred from intensive care to clinical care. All the patients completed 6MWT on the 1st and 4th day of the clinical care and PFT on the 4th day. The SF-36 was measured again when all patients included in the study visited the hospital for a check-up 6 weeks after the operation.

#### 2.5.1. Standard Care Program

In Turkey, cardiovascular surgery clinics have their own intensive care units. In these intensive care units, nurses who are specifically trained for this field and experienced work. Besides, physiotherapists generally do not work in in-patient clinics and intensive care units.

The standard care described in this section includes the care provided to patients undergoing CABG, as in all surgical patients in Turkey. This care includes deep breathing exercises, coughing exercises, tapotement, use of an incentive spirometer, and mobilization. The number of sessions and the repetitions of these applications vary according to the prescribing exercise given by the surgeons.

In this study, patients in the control group received a standard care program. This program is specific to the cardiovascular surgery clinic of the hospital where the study was conducted.

The control group patients were given training on the exercises to be performed after the surgery in their room the day before the surgery by the clinical nurses. These exercises are deep breathing exercises, coughing exercises, tapotement, use of incentive spirometry, and mobilization. The clinical nurses got the control group patients to perform the postoperative exercises. During the postoperative period, all patients receiving standard care performed deep breathing/coughing exercises and tapotement 4 times a day, the use of an incentive spirometer 10 times a day, and mobilization with an increasing distance each time (Table 1).

#### 2.5.2. Pulmonary Rehabilitation Program

The pulmonary rehabilitation program was designed by a physician (FY) with six years of cardiopulmonary rehabilitation experience. The intervention group patients that were admitted to the clinic for CABG surgery were briefed about the study protocol. The researcher (ZG) conducted the briefing in the rooms of the patients one day before the surgery. The researcher showed all the elements of the program and asked the patients to repeat the activities. The patients were observed, and the necessary corrections were made. This training lasted about 40 minutes.

This therapy consisted of mobilization, active exercises of the upper and lower extremities, and the chest physiotherapy (Table 1). Chest physiotherapy consisted of an active cycle of breathing techniques (controlled coughing and huffing techniques combined with diaphragmatic breathing and deep breathing exercises) and postural drainage. Pulmonary rehabilitation program was started with 1-2 METS exercises and gradually increased based on the tolerance and requirements of the patients. The exercise protocol consisted of progressive exercises as performed in standardized phase I cardiac rehabilitation and progressed from passive movements to walking on the first day after the surgery and finally climbing the stairs for two floors on the fourth day. The mobilization of the patients was managed as early as possible (within the first 24 hours) (in-bed range of motion exercise, sitting on the edge of the bed, standing up, and walking 30 meters). Walking distance has been increased gradually (according to the Modified Borg scale: 2-4) without causing excessive fatigue on the following postoperative days [38].

After CABG, patients with stable hemodynamics after extubation had pulmonary rehabilitation for 2 sessions/day on the first day and 3 sessions/day after transfer to the in-patient clinic until the patient was discharged.

After each session, each patient continued with an incentive spirometer after resting. During their stay in the in-patient clinic, the patients were allowed to work with an incentive spirometer in addition to the exercises.

A training booklet prepared by the researchers was given to the patients before discharge. The booklet included the information on how, when, and with how many repetitions these exercises should continue to be performed by the patients. The patients were also informed that these exercises should be repeated for 30 days following the discharge.

### 2.6. Data Analysis

Statistical analysis of the study data was carried out by using the SPSS 20.0 package program (SPSS Inc., Chicago, IL, USA) under the consultancy of the Department of Biostatistics. Descriptive statistics of continuous variables were shown with mean, standard deviation values, and descriptive statistics of categorical variables were shown with frequency and percentage. The normal distribution of the data was evaluated with the Shapiro-Wilk test. Independent group comparisons of the averages of APACHE II and EuroSCORE values were analysed through the independent samples *T*-test. The independent samples *T*-test and Mann–Whitney *U* test were used in the independent group comparisons of the PFT Parameters (FVC, FEV_1_, FVC/FEV_1_) and mental and physical component scores. The temporal differences for the PFT parameter scores and mental and physical component scores were analysed using the paired-samples test and Wilcoxon Signed Rank Test. Two-way Repeated Measures ANOVA was used to evaluate within- and between-group changes for total mean scores of Functional Capacity (6MWT) at three different time points. Cohen's *d* and *r* = (*Z*/(√*N*obs)) formulation were used to calculate the effect size for the comparison of PFT parameters and mental and physical component scores of independent groups. According to Cohen's definitions, *d* = 0.2 be considered a “small” effect size, 0.5 represents a “medium” effect size and, 0.8 a “large” effect size; *r* value intervals are defined as *r* = 0.1 to 0.3: small effect; 0.3 to 0.5: intermediate effect; 0.5 and higher: strong effect [39]. An alpha level of *p* < 0.05 was accepted as statistically significant.

### 2.7. Ethics Statement

Before the study, permission to perform the study was obtained from the Clinical Research Ethics Committee (2016/806). Written approval to carry out the study was also obtained from the hospital administration. Additionally, the patients included in the study were informed that the participation was voluntary based and this was put into writing with an informed consent form.

## 3. Results

A total of 102 patients undergoing CABG were considered for their eligibility. The study was completed with 25 patients in the intervention group and 25 patients in the control group as 46 patients did not meet the inclusion criteria, two of them did not want to participate, and four of them left the study after agreeing to participate (Figure 1).

### 3.1. Baseline Characteristics of Participants

The distribution of patients according to their sociodemographic characteristics and their risk factors is shown in Table 2. When the patients were assessed for their mortality risk, all the patients in both groups were in the low-risk group. There was not any significant difference between the groups when the average EuroSCORE values of the groups were compared (*p* = 0.226). Additionally, there was not any significant difference between the groups in terms of acute physiological score (*p* = 0.159), age assessment (*p* = 0.420), and APACHE II scores (*p* = 0.138) in the first 24 hours of postoperative intensive care (Table 3).

### 3.2. Primary Outcome: PFT and 6MWT

The preoperative mean baseline FVC value of the patients was found to be 89.96 ± 21.9 for the intervention group and 81.72 ± 15.5 for the control group. When the FVC mean values of the patients on the 4th day of clinical care were examined, the mean value of the patients in the intervention group (51.28 ± 14.9) was significantly higher with medium size effect than the mean value (42.76 ± 11.3) in the control group (*p* = 0.027; effect size (*d*) = 0.643). Besides, the FVC values of the patients in both groups on the 4th day of clinical care significantly decreased compared to the baseline FVC values (*p* < 0.001). The preoperative mean baseline FEV1 value of the patients was found to be 91.12 ± 20.6 for the intervention group and 82.56 ± 16.0 for the control group. When the FEV1 mean values of the patients on the 4th day of clinical care were examined, the mean value of the patients in the intervention group (53.16 ± 15.1) was significantly higher with medium size effect than the mean value (44.28 ± 11.5) in the control group (*p* = 0.024; effect size (*d*) = 0.658). Also, the FEV1 values of the patients in both groups on the 4th day of clinical care significantly decreased compared to the baseline FEV1 values (*p* < 0.001). The mean FVC/FEV1 values of the patients were determined to increase in both groups according to the baseline, and there was no significant difference between both groups and measurements (*p* > 0.05) (Table 4).

When the mean distance of 6MWD of the groups was examined, there was a significant difference between the groups (*p* (*G*) = 0.009). The walking distances of the intervention group were greater than the control group at all measurement times (preoperative, 1st and 4th day of clinical care). In both of the postoperative measurements (1st, 4th day), the 6MWD values in both groups were lower than the baseline values. However, the walking distances of the control group decreased more than the intervention group (*p* (*T*) < 0.001). Group-by-time interaction was not significant (*p* (*T*∗*G*) > 0.05) (Table 5).

### 3.3. Secondary Outcome: QoL

**Figure 2:**
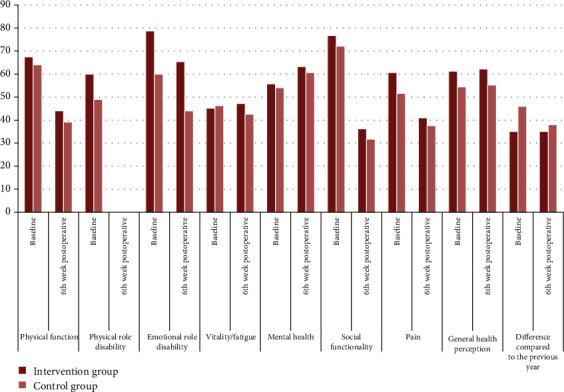
Comparison of groups according to the scores for the SF-36 subscale of quality of life.

The scores of the SF-36 scale performed at the 6th postoperative week were determined to decrease in both groups, and the mean scores of the intervention group were higher than the control group for almost all subdimensions of the scale. However, there was no significant difference between the groups (*p* = 0.015) (Figure 2).

The preoperative mean baseline PCS scores of the intervention group were found to be 69.50 (23.75-90) and 50.00 (15-92) for the control group. When the postoperative 6th week PCS scores of the patients were examined, the scores of the patients in the intervention group were (37.00 (25.75-46.25)) higher than those in the control group, (33.75 (18.75-43.25)). However, the difference was not significant (*p* > 0.05). Besides, the PCS scores of the patients in both groups on the postoperative 6th week significantly decreased compared to the baseline PCS values (*p* < 0.001).

The preoperative mean baseline MCS scores of the intervention group were found to be 67.25 (7.75-97.75) and 69.50 (3.25-89) for the control group. When the postoperative 6th week MCS scores of the patients were examined, the scores of the patients in the intervention group were (60.50 (23.25-74.50)) higher than those in the control group, (43.00 (19.25-68.50)). However, the difference was not significant (*p* > 0.05). Besides, although a decrease was observed in both groups at postoperative 6th week (*p* = 0.001, *p* = 0.007), the decrease was less in the intervention group (Table 6).

## 4. Discussion

Pulmonary dysfunction is a major complication that leads to a significant decrease in lung volumes, vital capacity, and oxygenation, and a 30%-50% decrease in FEV1, FVC, and functional residual capacity [13]. It is indicated in the literature that FEV1 and FVC values play a role in identifying the presence of dysfunction in the lung and obstruction in major airways [40]. Pulmonary complications after the on-pump cardiac surgery are common conditions and known to affect mortality and morbidity [41]. Pulmonary physiotherapy in the preoperative and postoperative care of patients undergoing cardiac surgery is very important in terms of preventing pulmonary complications, offering a better prognosis for the patient, and its contributions to the treatment [42]. In this study, in which the effect of PR applied to patients undergoing CABG surgery on respiratory functions of patients was examined, FEV1 and FEVC values in the group were significantly decreased on the 4th day of clinical care compared to the baseline [25, 27, 43].

6MWT is a simple, safe, and reliable test that can identify changes in the walking capacity of patients receiving physiotherapy after the surgery [27]. It was stated in Cordeiro et al.'s (2016) study, which investigated the effect of respiratory muscle exercises in patients undergoing cardiac surgery, which the walking distance on the postoperative 3rd day of the patients in the exercise group was a lot longer than the patients, who did not exercise [44]. In another study in which active breathing exercises were applied to one group of patients, and an incentive spirometer to the other group, the change was less in the 6MWT walking distance of the patients in the group with active breathing exercises preoperatively and postoperatively on the 5^th^ day [45]. Savcı et al. (2011) reported in their study that short-term inspiratory muscle training after CABG surgery resulted in a clinically significant change in six-minute walking distance scores [27]. The findings of our study are consistent with the literature. The decrease in walking distance of the patients on the first day of clinical care is believed to be the result of the early postoperative period and the increase on the 4^th^ day of clinical care as a result of the recovery period. However, the fact that the walking distance change rate in the intervention group is lower than the control group may be related to the PR program.

CABG surgery is aimed at improving health-related QoL, especially in terms of mental and physical functions [46], and prolonging life [47]. However, the literature states that CABG can affect patients' QoL. Pulmonary rehabilitation, in addition to increasing oxygen consumption and exercise capacity, improves the QoL of patients undergoing cardiac surgery [48]. In this study, the QoL of patients was measured 6 weeks after CABG surgery. There was a decrease in almost all subdimensions of the scale in both groups, albeit less in the PR group. Also, both PCS and MCS scores in both groups decreased compared to the preoperative period values. After CABG, the QoL of the patients has been reported to decrease in the early postoperative period [49]. Valkenet et al. (2017), as a result of the study in which they examined the effect of inspiratory muscle exercises on QoL, reported that there was no improvement in quality of life scores [50]. Pačarić et al. (2020) revealed that one month after CABG, the QoL increased, however, it was still insufficient [51]. In the literature, most of the improvement in both physical and mental component summary of QoL has been reported to occur in the first 6 months in CABG patients [52, 53]. Our study results showed that the QoL of CABG patients decreased in the early postoperative period, and the effects of the PR program were positive in the short term, but not sufficient.

### 4.1. Limitation

The sample of the study consisted of Turkish patients who underwent CABG surgery in the cardiovascular surgery clinic of a private hospital in western Turkey. These findings cannot be generalized to all CABG patients. Furthermore, since the PR program was designed specifically for these patients in this study, the results cannot be generalized to all CABG patients. Although this study indicates that PR can help patients regain their functional capacity more quickly and may have positive effects on the QoL in the short term, these findings cannot be generalized to all CABG patients. Since the study was a Master's Thesis, a second observer could not be included for evaluations. The fact that the same researcher applied PR and collected the data in face-to-face interviews with patients without the help of a second observer might be considered as the main limitation of the study. Also, the fact that the researchers were not blinded to the intervention conditions may have affected the result due to small biases. Another limitation of the study was that the respiratory functions of the patients were measured only with PFT, and radiological imaging could not be performed for the evaluation of atelectzaic areas in the lung due to the limited budget. Concerns about the limited time available during the study prevented us from investigating the long-term effects of PR on pulmonary functions and QoL. Besides, only patients in a low-risk group were evaluated in this study. We recommend that future studies be conducted with patients in a larger population and with high-risk level patients and that these studies also investigate the long-term effects of PR on QoL.

## 5. Conclusion

The results of this study revealed that PR applied to patients undergoing CABG helped patients recover their functional capacity faster. The short-term effects of PR on the QoL of patients undergoing CABG were also positive.

## Figures and Tables

**Table 1:** Standard care and PR program.

Standart Care Program (Control Group)	
Preoperative training (1 day before surgery)	(i) Deep breathing exercise (ii) Coughing exercise (iii) Tapotement (iv) Incentive spirometer (v) Mobilization
Postoperative (ICU and Clinic)	(i) Deep breathing exercises 10 repetition / 4 sessions (ii) Coughing exercises 10 repetition / 4 sessions (iii) Tapotement 10 repetition / 4 sessions (iv) Incentive spirometer 10 repetition / 10 sessions (v) Mobilization (increasing distance each time)

Pulmonary Rehabilitation Program (Intervention Group)	
Preoperative training (1 day before surgery)	(i) Active Cycle of Breathing Techniques (ii) Postural drainage (iii) Lower-upper extremity exercises (iv) Mobilization
ICU	(i) Active Cycle of Breathing Techniques 10 repetition/ 2 sessions (ii) Postural drainage 1 repetition / 2 sessions (iii) Lower-upper extremity exercises 10 repetition/ 2 sessions (iv) Active extremity exercises; actively turning the patient in bed, mobilizing the patient out of bed with a portable ventilator, sitting by the bed, moving to the chair after sitting at the bedside, standing, standing on the side of the bed and standing upright, transferring to the chair) (37) (v) Mobilization (stood up and walked 30 meters and gradually increased walking distance)
Clinic	(i) Active Cycle of Breathing Techniques 10 repetition/ 3sessions (ii) Postural drainage 1/3 sessions (iii) Lower-upper extremity exercises 10 repetition/ 3 sessions Day 1: extremity exercises, sitting at the bedside-sitting on the chair Day 2: extremity exercises, sitting at the bedside-sitting on the chair, shoulder-neck exercises, Day3: extremity exercises, sitting at the bedside-sitting on the chair, shoulder-neck exercises, standing hip and knee exercises, light squatting exercises Day4: extremity exercises, sitting at the bedside-sitting on the chair, shoulder-neck exercises, standing hip and knee exercises, light squatting exercises, climbing stairs (iv) Mobilization (walking distance increased gradually everyday)

**Table 2:** Distribution of sociodemographic characteristics and smoking status of patients.

Characteristics	Intervention (PR) group (*n* = 25)		Control group (*n* = 25)	
	*n*	%	*n*	%
*Gender*				
Female	8	32	11	44
Male	17	68	14	56
*Marital status*				
Married	23	92	21	84
Singles	2	8	4	16
*Education status*				
Illiterate	3	12	7	28
Primary school	18	72	14	56
High school	1	4	4	16
University	3	12	—	—
*Professional status*				
Retired	10	40	9	36
Housewife	7	28	11	44
Farmer	4	16	2	8
Civil servant	2	8	1	4
Other	2	8	2	8
*Smoking status*				
Nonsmoker	9	36	13	52
Ex-smoker	8	32	10	40
Smoker	8	32	2	8
	Mean ± SD		Mean ± SD	
Age (years)	60.36 ± 8.3		61.96 ± 7.5	
Smoking duration (years) (mean ± SD)	29.93 ± 12.6		21.75 ± 12.2	
Cigarette usage per day (PCs) (mean ± SD)	18.12 ± 12.6		16.83 ± 10.6	

SD: standard deviation.

**Table 3:** Comparison of groups according to the EuroSCORE and first 24 h intensive care APACHE II scores.

	Intervention (PR) group (*n* = 25)	Control group (*n* = 25)	*p* ^∗^
	Mean ± SD	Mean ± SD	
EuroSCORE	1.00 ± 1.0	1.36 ± 0.9	0.226
Apache II	11.32 ± 2.2	12.48 ± 3.1	0.138
Acute physiological scoring	5.76 ± 1.4	6.60 ± 2.5	0.159
Age evaluation	3.40 ± 1.3	3.72 ± 1.4	0.420

SD: standard deviation; ^∗^*p* < 0.05, independent samples *t* test.

**Table 4:** Comparison of total mean scores of PFT parameters (FVC, FEV_1_, FVC/FEV_1_) in intervention (PR) and control groups.

	Intervention (PR) Group	Control Group	Test results	p value	Effect size (*d*)	95% Confidence interval	
	Mean ± SD	Mean ± SD				Lower	Upper
*FVC, % predicted*							
Preoperative baseline	89.96±21.9	81.72±15.5	^∗^*t* = −1.532	0.132	-0.433	-0.136	0.997
4^th^ Day of Clinical Care	51.28±14.9	42.76±11.3	^∗^*t* = −2.275	0.027	-0.643	0.072	1.210
p value	*p* < 0.001	*p* < 0.001					
Test results	^∗∗^*t* = 12.321	^∗∗^*t* = 15.144					
*FEV_1_, % predicted*							
Preoperative baseline	91.12±20.6	82.56±16.0	^∗^*t* = −1.634	0.109	-0.462	-0.100	1.023
4^th^ Day of Clinical Care	53.16±15.1	44.28±11.5	^∗^*t* = −2.327	0.024	-0.658	0.088	1.228
p value	*p* < 0.001	*p* < 0.001					
Test results	^∗∗^*t* = 11.732	^∗∗^*t* = 14.017					
*FEV_1_/FVC, % predicted*							
Preoperative baseline	107.52±10.4	108.56±12.3	^∗^*t* = 0.321	0.749	0.091	-0.645	0.463
4^th^ Day of Clinical Care	109.88±10.4	109.08±14.3	^∗^*t* = −0.225	0.823	-0.064	-0.490	0.618
Test results	^∗∗^*t* = −0.979	^∗∗^*t* = −0.149					
p value	*p* < 0.337	*p* < 0.883					

PR: pulmonary rehabilitation; PFT: pulmonary function test; FEV1: challenging expiratory volume; FVC: challenging vital capacity; ^∗^independent samples *t*-test; ^∗∗^paired samples *t*-test *p* < 0.05.

**Table 5:** Comparison of total mean scores of Functional Capacity (6MWT) in intervention (PR) and control groups.

	Intervention (PR) group	Control group	*p* value (*G*)	*p* value (*T*)	*p* value (*T*^∗^*G*)
	Mean ± SD	Mean ± SD			
6MWT, m			0.009	<.001	0.166
Preoperative baseline	270.00 ± 66.3	217.00 ± 76.7			
1^th^ day of clinical care	107.00 ± 53.3	82.40 ± 49.0			
4^th^ day of clinical care	177.80 ± 58.5	137.40 ± 43.2			

6MWD: 6-Minute Walk Test; *p* (*G*): significance between groups; *p* (*T*): significance between time points; and *p* (*T*∗*G*), significance of time ∗ group interaction.

**Table 6:** Measurements of mental and physical component summary on for patients in all groups.

	Intervention (PR) Group	Control Group	Test results	*p value*	Effect size (r)^a^	95% Confidence interval	
	Median (Min-Max)	Median (Min-Max)				Lower	Upper
*PCS*							
Preoperative baseline	69.50 (23.75-90)	50.00 (15-92)	^∗^*Z* = −0.980	0.327	0.138	0.102	0.387
6^th^ Postoperative Week	37.00 (25.75-46.25)	33.75 (18.75-43.25)	^∗^*Z* = −1.923	0.054	0.271	0.062	0.495
Test results	^∗∗^*Z* = −3.916	^∗∗^*Z* = −3.700					
*p value*	*p* < 0.001	*p* < 0.001					
*MCS*							
Preoperative baseline	67.25 (7.75-97.75)	69.50 (3.25-89)	^∗^*Z* = 0.796	0.426	0.112	0.140	0.359
6^th^ Postoperative Week	60.50 (23.25-74.50)	43.00 (19.25-68.50)	^∗^*Z* = −1.688	0.091	0.238	0.000	0.451
Test results	^∗∗^*Z* = −3.337	^∗∗^*Z* = −2.704					
*p value*	*P* = 0.001	*P* = 0.007					

PCS: SF36 Physical Component Summary; MCS: SF36 Mental Component Summary; ^∗^Mann–Whitney *U* test, ^∗∗^Wilcoxon Signed Rank Test. ^a^*r* ( = *Z*/(√*N*obs)).

## Data Availability

The datasets used and/or analysed during the current study are available from the corresponding author upon reasonable request.
